# Intra-guild competition and its implications for one of the biggest terrestrial predators, *Tyrannosaurus rex*

**DOI:** 10.1098/rspb.2010.2497

**Published:** 2011-01-26

**Authors:** Chris Carbone, Samuel T. Turvey, Jon Bielby

**Affiliations:** 1 Institute of Zoology, Zoological Society of London, Regent's Park, London NW1 4RY, UK; 2 Department of Infectious Disease Epidemiology, Imperial College London, St. Mary's Hospital, Norfolk Place, London W2 1PG, UK

**Keywords:** Scavenging, hunting behaviour, scramble competition, *Tyrannosaurus rex*

## Abstract

Identifying tradeoffs between hunting and scavenging in an ecological context is important for understanding predatory guilds. In the past century, the feeding strategy of one of the largest and best-known terrestrial carnivores, *Tyrannosaurus rex*, has been the subject of much debate: was it an active predator or an obligate scavenger? Here we look at the feasibility of an adult *T. rex* being an obligate scavenger in the environmental conditions of Late Cretaceous North America, given the size distributions of sympatric herbivorous dinosaurs and likely competition with more abundant small-bodied theropods. We predict that nearly 50 per cent of herbivores would have been within a 55–85 kg range, and calculate based on expected encounter rates that carcasses from these individuals would have been quickly consumed by smaller theropods. Larger carcasses would have been very rare and heavily competed for, making them an unreliable food source. The potential carcass search rates of smaller theropods are predicted to be 14–60 times that of an adult *T. rex*. Our results suggest that *T. rex* and other extremely large carnivorous dinosaurs would have been unable to compete as obligate scavengers and would have primarily hunted large vertebrate prey, similar to many large mammalian carnivores in modern-day ecosystems.

## 1. Introduction

Scavenging behaviour is associated with a diverse range of important ecological and behavioural processes including kleptoparasitism [1], disease transmission [2], species distributions [3–5] and nutrient cycling [6], but despite its importance it remains a poorly understood phenomenon [7]. Most carnivorous species today rely on a combination of hunting and scavenging [7,8], although some groups of species subsist almost exclusively on scavenging [9]. Effective scavengers must possess high movement speeds but low locomotion costs, and the ability to detect carcasses over long distances in order to outcompete other scavengers and predators [10]. These factors explain why vultures are such highly successful obligate scavengers, while sit-and-wait predators (e.g. viperid snakes) are reliant almost exclusively on hunting [7]. However, many physical features that help species scavenge successfully (e.g. powerful build; sharp teeth; fast speed; good vision and sense of smell) are also useful for hunting. As a result, it can be difficult to diagnose feeding strategies of species from their physical characteristics alone, especially for extinct carnivores known only from the fossil record [11–13].

In the past century, the feeding strategy of one of the best known and largest terrestrial carnivores, *Tyrannosaurus rex*, has been the subject of considerable debate and attention (e.g. [14–20]). Proposed support for the species being a primary or obligate scavenger includes its disproportionately tiny forelimbs and apparently reduced eyes [16], enlarged olfactory bulbs [21], and large body size and upright stance as a possible adaptation for finding carcasses, although the latter two traits may also have been an advantage for locating live prey [15]. However, enlarged olfactory bulbs may be associated with behaviours unrelated to food acquisition [22,23], and the eyes of *T. rex* are in fact large in both relative and absolute terms [24]. The binocular vision, bite force and impact-resistant teeth of *T. rex* suggest instead that it may have been better adapted for an active predatory lifestyle [18,20,25,26].

Nearly all of the debate over the feeding ecology of *T. rex* has focused on the interpretation of functional morphology rather than on energetic or ecosystem-level considerations. A recent attempt to determine the feasibility of *T. rex* being an obligate scavenger addressed the energetic costs of movement and the amount of meat available from carcasses [17]. Based on reasonable assumptions of energetic rates, travel speeds and carcass detection ability, this study concluded that *T. rex* could feasibly have survived as an obligate scavenger, although energetically it might have been a marginal strategy. However, as yet no analyses of the feeding ecology of *T. rex* have investigated wider patterns of theropod and other dinosaurian diversity in Late Cretaceous faunas [27], in particular, the impacts of competition from other sympatric carnivorous theropods, or the size distribution of carcasses available to potential scavengers of that period.

Most extant carnivore guilds contain a number of competing predatory/scavenging species [28], and the Late Cretaceous North American dinosaur fauna also consisted of several small-bodied non-avian carnivorous theropods in the Tyrannosauridae, Oviraptorosauria (Caenagnathidae), Deinonychosauria (Dromaeosauridae and Troodontidae) and Coelurosauria incertae sedis [27]. Although oviraptorosaurs and troodontids may not have been obligate carnivores [29,30], many of these sympatric theropods were largely or completely carnivorous, and there is evidence for both active predation and late-stage carcass consumption (probable scavenging) by dromaeosaurs [31]. In modern-day ecosystems, carcasses are typically a rare, widely dispersed and unpredictable resource, and competition among scavengers is often seen as a form of scramble competition [7]. If the same conditions applied in Late Cretaceous ecosystems, *T. rex* could have been in competition for resources with a range of different-sized theropods, also including juvenile and sub-adult *T. rex* individuals, which may have been relatively abundant compared with adults [32]. In our analysis, we focus on Late Cretaceous theropod species weighing 16–20 kg and up to the size of *T. rex*. Our lower size range was based on evidence from terrestrial mammalian carnivore guilds, because species of this weight range (e.g. wild dogs, jackals, hyenas) are extremely effective hunter/scavengers [8] (table 1). If the smaller, more abundant theropod species and *T. rex* age classes were therefore also primarily hunters that scavenged opportunistically, this might have placed extreme pressure on the resources available from scavenging in the Late Cretaceous theropod community.

**Table 1.** Species and body masses of carnivorous non-avian theropod dinosaurs of Late Cretaceous North America. Midpoint taken when a range of body mass estimates is given in the literature. *Richardoestesia* is only known from jaws and teeth; since maximum tooth size is slightly smaller than that of *Saurornitholestes* [33], we estimate a body mass of *ca* 20 kg for this genus based on *Saurornitholestes* body mass estimates. Body mass estimates for *Albertosaurus sarcophagus* and *T. rex* taken as mean of all measurements for adult individuals that had reached somatic maturity given in Erickson *et al*. [34]. *Nanotyrannus* has been interpreted as a possible juvenile *Tyrannosaurus* by some authors [35], but we provisionally retain it here as a valid taxon following [27]. Mass categories and the estimated percentage that each size category contributes to the total carnivore guild are also presented (figure 1).

species	family	mass (kg)	mass categories, kg (with estimated %)	reference
*Dromaeosaurus albertensis*	Dromaeosauridae	16	20.6 (79.9%)	[36]
*Richardoestesia gilmorei*	Coelurosauria incertae sedis	20		see legend
*Richardoestesia isosceles*	Coelurosauria incertae sedis	20		see legend
*Saurornitholestes langstoni*	Dromaeosauridae	23		[37]
*Velociraptor* sp.	Dromaeosauridae	24		[38]
*Troodon formosus*	Troodontidae	50	58.3 (19.0%)	[39]
*Chirostenotes elegans*	Caenagnathidae	63		[40]
*Chirostenotes pergracilis*	Caenagnathidae	63		[40]
*Nanotyrannus lancensis*	Tyrannosauridae	1100	1123 (0.9%)	[39]
*Albertosaurus sarcophagus*	Tyrannosauridae	1146		[34]
*Tyrannosaurus rex*	Tyrannosauridae	5347	5347 (0.1%)	[34]

Interpreting patterns of relative abundance is notoriously difficult from the fossil record, due to taphonomic biases typically favouring the preservation of large-bodied species [41]. Skeletons of small-bodied Late Cretaceous dinosaurs are very rare and new species continue to be discovered, whereas the remains of large-bodied dinosaurs typically dominate Late Cretaceous fossil deposits [38,42]. However, even though they are recognized to have a much lower preservation potential, the observed fossil abundance of small putatively carnivorous theropods (dromaeosaurs and troodontids) is similar to that of tyrannosaurids in North American Late Cretaceous deposits [42], and many of these deposits show a relatively high abundance of both small theropods and small herbivorous dinosaurs (hypsilophodontids and pachycephalosaurs) [43]. This suggests that smaller carnivores and herbivores were proportionally much more abundant than tyrannosaurids in Late Cretaceous ecosystems. The abundance of small species within dinosaur guilds would be supplemented by the presence of earlier life stages of larger species. Body mass abundance patterns in fishes, another vertebrate group where individuals are independent before adulthood, are similar to those found in mammals when one considers the overall size classes rather than species weight classes [44]; assumptions about relationships between abundance and size at a faunal level are therefore likely to be robust to changes in the relative abundance of some of the smaller species.

In this paper, we explore the potential competitive ability of *T. rex* as an obligate scavenger. We take into account the presence of other carnivorous theropod individuals in Late Cretaceous ecosystems, and the estimated spatial distribution of carcasses of different herbivorous dinosaur species that would be available for scavengers. We make the assumption that, by analogy with vertebrate assemblages in modern-day terrestrial ecosystems, many predators are also opportunistic scavengers, so that there are more potential consumers of carcasses than carcasses available for these predatory guilds. We assess the likelihood that adult *T. rex* could have been an obligate scavenger based on expected carcass encounter rates for this species compared with those for sympatric smaller theropod dinosaurs. We follow Ruxton & Houston [17] in drawing explicitly on modern ecological analogues, by basing our analysis on the production rate of large carcasses estimated from the terrestrial large mammal community of the well-studied modern-day Serengeti ecosystem [9]. We also base our predictions of relative abundance and travel speed on well-established body size abundance and speed relationships found in terrestrial mammals [45–47].

## 2. Methods

Lists of non-avian dinosaur species that were stratigraphically coeval and geographically sympatric with *T. rex* and which could potentially act as competitors or prey were collected from records of Late Cretaceous (late Campanian to late Maastrichtian) fossil formations in North America [27]. Only those dinosaur taxa that were positively identified to species or genus level from formations that also contained tyrannosaurid fossils identified as *T. rex* were categorized in our study as being sympatric; all provisional or tentative species or genus identifications were interpreted as being definite identifications for the purposes of analysis. Body mass estimates for these dinosaurs were obtained from the scientific literature (tables 1 and 2). We exclude the herbivorous ornithomimosaurs [54] from our analyses of carnivorous theropods but include caenagnathid oviraptorosaurs and troodontids, following earlier studies [55–57]. Although there may have been minor variations in dinosaur faunal composition across the geographical range of *T. rex* during the Late Cretaceous (e.g. titanosaurid sauropods were restricted to the southwestern United States [27]), our species list is treated as representing a consistent sympatric faunal unit across this region for the purposes of analysis. A slightly different species list is also available on the Paleobiology Database (http://paleodb.org/cgi-bin/bridge.pl). However, as our analysis of theropod performance focuses on a comparison between the largest and smallest theropod size classes (see below), it is robust to such minor changes in faunal composition.

**Table 2.** Species and body masses of herbivorous dinosaurs of Late Cretaceous North America. Ornithischian and sauropod body masses taken from the midpoint of genus-level estimates in Peczkis [48]; ornithomimosaur body masses taken from Christiansen [37]. Species diversity from Weishampel *et al*. [27] revised following [49–52]; *Dyslocosaurus polyonychius* also excluded as the suggested Late Cretaceous occurrence of this species is probably erroneous [53]. Mass categories and the estimated percentage each category contributes to the total herbivore guild are based on herbivore mass–abundance relationships found in extant mammalian herbivores (see also figure 1).

species	family	body mass (kg)	mass categories, kg (with estimated %)
*Parksosaurus warreni*	Hypsilophodontidae	55	75 (49.3%)
*Prenocephale edmontonensis*	Pachycephalosauridae	85	
*Ornithomimus velox*	Ornithomimosauridae	155	216 (36.8%)
*Struthiomimus* sp.	Ornithomimosauridae	175	
*Thescelosaurus garbanii*	Hypsilophodontidae	250	
*Thescelosaurus neglectus*	Hypsilophodontidae	250	
*Leptoceratops gracilis*	Leptoceratopsidae	250	
*Montanoceratops* sp.	Leptoceratopsidae	550	700 (6.0%)
*Pachycephalosaurus wyomingensis*	Pachycephalosauridae	850	
*Edmontosaurus annectens*	Hadrosauridae	2500	2500 (6.7%)
*Edmontosaurus regalis*	Hadrosauridae	2500	
*Edmontosaurus saskatchewanensis*	Hadrosauridae	2500	
*Lambeosaurus* sp.	Hadrosauridae	2500	
*Parasaurolophus walkeri*	Hadrosauridae	2500	
*Edmontonia rugosidens*	Nodosauridae	2500	
*Ankylosaurus magniventris*	Ankylosauridae	5500	5000 (0.6%)
*Triceratops horridus*	Ceratopsidae	8500	8500 (0.4%)
*Alamosaurus sanjuanensis*	Saltasauridae	25 000	25 000 (0.2%)

We based our estimates of carcass production on data from the Serengeti ecosystem [17]. We assume that this is a valid ecological comparison, because although ecosystems in the Late Cretaceous may have been more productive and contained more large-bodied species than terrestrial mammalian systems today, they would also be expected to contain a higher abundance of predators, and the fossil record of large-bodied species contains similar predator–prey ratios to those recorded in modern-day ecosystems [58].

Our approach relies on the use of allometric equations to estimate how factors such as abundance and search capacity vary with body mass. Many biological characteristics of animals are related to body mass and can be described with power equations taking the form of *Y* = *a* × *M*^*b*^, where *a* is a constant, *M* is mass (kg) and *b* represents the allometric exponent, which influences the relative change in the value *Y* with changes in body mass. These equations are commonly used in multi-species studies on the influence of body mass on species characteristics [59].

We assume that population densities of herbivorous and theropod dinosaurs, *N*_h_ and *N*_t_ (number of individuals km^−2^), are related to the following power equations:
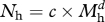
and
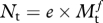
where *M*_h_ and *M*_t_ are in kilograms, *c* and *e* are constants corresponding to the number of 1 kg animals km^−2^, and *d* and *f* are scaling factors.

**Figure 1.**
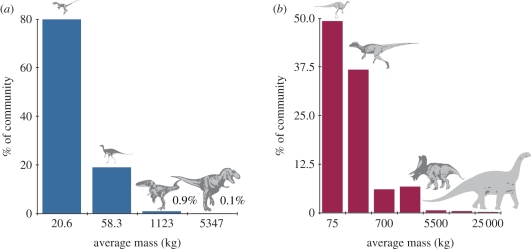
Body mass categories and predicted relative abundances of (*a*) non-avian carnivorous theropods and (*b*) herbivorous dinosaurs found in Late Cretaceous North America. Relative abundances were estimated from equations derived from abundance–mass relationships found in extant mammalian carnivores and herbivores [47,60] and from body masses for sympatric dinosaurs from the Late Cretaceous *T. rex* community (tables 1 and 2).

To estimate the relative numbers of Late Cretaceous herbivorous dinosaurs, we used values of *c* = 100 and *d* = −0.75, similar to the values estimated from data on herbivorous mammal abundance [45]. Estimates of carnivorous theropod dinosaur abundances were derived from mammalian carnivore data [47], where *e* = 1.97 and *f* = −0.88. For the purposes of illustration, herbivorous and carnivorous dinosaur species, including different age classes of the larger species, were then grouped into seven and four size classes, respectively, corresponding to the natural body size groupings shown by the data (tables 1, 2 and figure 1).

To explore how the presence of sympatric competing theropods affects the likelihood of adult *T. rex* being an obligate scavenger, we used estimates of carcass encounter rate following the model proposed by Ruxton & Houston [17]. Our approach, however, differs from Ruxton & Houston in that we focus on estimating relative encounter rates of theropod scavengers accounting for size-dependent differences in estimated population density, travel speed and search capacity in relation to the size and likely distribution of available carcasses. Also unlike Ruxton & Houston, we do not explicitly attempt to estimate intake rates of a scavenging *T. rex* but focus our analysis on the relative search areas of an adult *T. rex* compared with other competing species and age classes. Following Ruxton & Houston, we assumed that the forager moves along a path where the search area is defined by the speed *S*_t_ (m s^−1^) and detection distance *D*_t_. Because we also need to estimate how these factors vary with body mass, we used the following allometric equations:
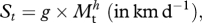
where *g* and *h* represent the constant and exponent for speed, respectively, and:
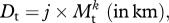
where *j* and *k* represent the constant and exponent for detection range, respectively.

In order to explore the potential impact of scavenging on carcasses, we calculated the total potential impact of the populations of different theropod species on carcass feeding by multiplying estimated population sizes by individual search rates (see above).

To estimate the population search rate *NA*_tot_ (km^2^ d^−1^) would be:
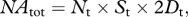
which expands to:
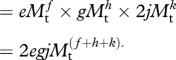


The constants *e*, *g* and *j* in this equation do not affect the relative impacts of scavenging by different theropods, but the scaling exponents have a critical influence on these relative values. Therefore, in order to compare these scanning rates we do not need to know the absolute values of the constants, which is useful as we do not have a good understanding of the absolute values of abundance, speed and detection range for any dinosaur species.

We initially chose variables for the constants and exponents to match Ruxton & Houston's [17] predictions for *T. rex* (table 3, scenario 1), but also used alternative values based on more extreme assumptions about relative differences between theropod species (table 3, scenarios 2–5). In scenario 1, we set the speed constant *g* = 11.9 and the speed scaling factor *h* = 0.05. We set the detection range constant *j* = 0.034 and the detection range scaling factor *k* = 0.1. These values predict a daily distance for an individual *T. rex* of 18.3 km and a detection distance of 80 m (approximately the values estimated in [17]), compared with 13.8 km d^−1^ and a 50 m detection range for a 20 kg theropod. We increased the exponents for the scaling of both speed and/or detection range with mass (scenarios 2–4) to increase the relative speed and detection distance for *T. rex*. Here we used combinations of values of 0.16 and 0.25 for the speed and detection range scaling factors *h* and *k*, respectively (broadly similar to values described in scaling studies on mammals [46,60]; table 3). For the purposes of illustration, we calculated an extreme case (scenario 5) where *T. rex* performs at the same level as smaller theropod species, with scaling factor values *h* = 0.35 and *k* = 0.54.

**Table 3.** Sensitivity analysis of the impact of varying speed and detection range for ‘small’ (a hypothetical 20 kg species) and ‘large’ (*T. rex*) theropod dinosaur populations. Scenario 5 shows extreme scaling where *T. rex*-sized theropods would be predicted to be able to cover the same areas as all other theropod species. In this case the constant *sc* was set to 6.6 to reduce the km d^−1^ estimate for *T. rex*.

	speed		detection		theropod mass (kg)	km d^−1^	*S*_t_ (km h^−1^)	*D*_t_ (km)	ratio^a^
scenario	const. *g*	exp. *h*	const. *j*	exp. *k*					
1	11.9	0.05	0.034	0.1	20	13.8	1.2	0.05	60.1
					5347	18.3	1.5	0.08	
2	11.9	0.16	0.034	0.1	20	19.2	1.6	0.05	32.5
					5347	47.0	3.9	0.08	
3	11.9	0.05	0.034	0.25	20	13.8	1.2	0.07	26
					5347	18.3	1.5	0.29	
4	11.9	0.16	0.034	0.25	20	19.2	1.6	0.07	14
					5347	47.0	3.9	0.29	
5	6.6	0.35	0.034	0.54	20	18.8	1.6	0.17	1.0
					5347	133.2	11.1	3.50	

^a^Ratio of the daily area covered by members of a species of 20 kg over that predicted for the *T. rex* population, based on an estimated density of 177.4 per 1000 km^2^ for the small species and 2.9 per 1000 km^2^ for *T. rex* (see text for details).

## 3. Results

Based on size–abundance relationships found in modern-day herbivorous mammals [45], we predict that just under 50 per cent of herbivorous carcasses in the *T. rex* community would have been in the 55–85 kg size range (figure 1). A productive modern-day ecosystem such as the Serengeti generates 4.38 kg of carcasses km^−2^ d^−1^ [9]. If we can assume that levels of carcass abundance were similar in Mesozoic terrestrial ecosystems, a substantial number of dinosaur carcasses would therefore have been present in Late Cretaceous North America, enough to sustain an individual adult *T. rex*. However, it becomes clear that finding these carcasses becomes a problem if we estimate the distributions of different-sized carcasses given this predicted rate of supply. Using the simplified assumption that all size classes of species produce carcasses at the same rate [47], we can easily calculate an expected distribution based on the Serengeti production rate as an even fraction of the number of herbivorous dinosaur size classes. If every carcass lasted 7 days before becoming consumed (or 14 days at 50% of the original body mass), most carcasses would be very widely dispersed indeed: on average there would be just one 75 kg carcass every 17 km^2^, one 700 kg carcass every 160 km^2^, and one 5 tonne carcass every 1000 km^2^, with a 25 tonne carcass every 5000 km^2^. Clearly, these carcasses would be difficult for individual scavengers to find without travelling great distances.

**Figure 2.**
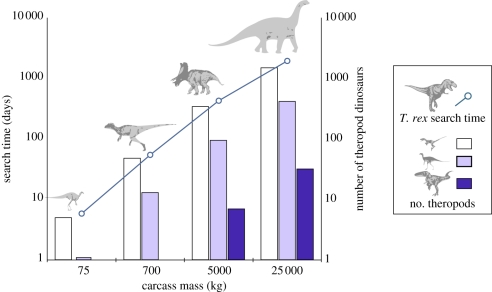
Left axis: the average time an individual theropod weighing just over 5 tonnes takes between individual encounters with carcasses of varying mass (70 kg to 25 tonnes) given expected carcass densities. Right axis: the number of competing theropods expected to arrive at the carcass in the time it takes *T. rex* to reach it. The *y* axes are represented on a log scale to allow the assessment of smaller values.

Based on Ruxton & Houston's calculations [17], we expect an adult *T. rex* to have covered just under 3 km^2^ a day (18.3 km daily distance × 2 × 0.08 km detection distance). Using our estimates of carcass distributions described above, we predict that *T. rex* would have been able to find a 75 kg carcass on average in just under 6 days, and a 700 kg carcass in approximately 55 days; on average, it would have had to search for over a year to find a 5 tonne carcass and five times that to find a 25 ton carcass (figure 2). The potential absence of titanosaurid sauropods, the largest size class of herbivorous dinosaurs, from part of the geographical range of *T. rex* [27] therefore has little bearing on the availability of carcasses in Late Cretaceous ecosystems.

How might populations of other smaller carnivorous theropod dinosaurs have impacted these carcasses during the time *T. rex* spent searching for them? Given the much higher predicted relative abundances of smaller theropod species and age classes compared with an adult *T. rex*, one might expect individuals of these species to have had a considerable effect on the amount of meat supplied by carcasses. In figure 2, we show the average expected numbers of theropod dinosaurs that would arrive at a carcass before an adult *T. rex* could reach it. The smaller, more abundant species would be expected to take less time to locate and arrive at a carcass. For a 75 kg carcass, we predict that on average five *Dromaeosaurus*-sized individuals and one *Troodon*-sized individual would already have arrived first, and would be expected to quickly consume most or all of the carcass. Larger carcasses would have attracted more theropods, and again would probably have been fully consumed before the arrival of *T. rex*. Overall for the *T. rex* community, we predict that the smallest carnivorous theropods collectively would have had carcass search rates over 60 times higher than adult *T. rex* under a model of modest scaling exponents for travel speed and detection range (i.e. small differences between theropods), and between 26–33 times higher given moderate scaling exponents (table 3). Only if we assume extreme scaling exponents, where an adult *T. rex* travelled over 130 km d^−1^ and had a detection range of around 3.5 km, could it have had the same search rate as smaller theropod species (table 3). However, even under these extreme and implausible parameters, competition would have been intense across the Late Cretaceous theropod guild. Although we do not show these calculations, if an adult *T. rex* had been even larger than our body mass estimate and instead nearer to 7 tonnes [34] following alternative estimates, we would expect approximately 30–73-fold lower search rates in this species given conditions in scenarios 1–3.

## 4. Discussion

Our results highlight the importance of taking both intraspecific and interspecific competition and the distribution of food resources into account when investigating the ecological viability of alternative carnivore foraging strategies. Ultimately, the nutritional ecology of a carnivore will be determined by the costs and benefits of different available strategies. Most terrestrial mammalian carnivores are primarily hunters, but scavenge opportunistically when carcasses become available [7]. A heavy reliance on scavenging will only be viable if this strategy can provide a reliable source of food. While our analysis does not examine these costs/benefits in relation to hunting at an individual level, we clearly show that given the distribution of carcasses and the potential for competition with other members of the carnivorous theropod guild, it is extremely unlikely that an adult *T. rex* could use scavenging as a long-term sustainable foraging strategy. The simple reason for this lies in the predicted overwhelming abundance of smaller-bodied competing theropods, with the smallest of these in the 16–25 kg range estimated to represent approximately 80 per cent of individuals in the guild (table 2).

In modern-day carnivore guilds, groups of African wild dogs (*Lycaon pictus*), wolves (*Canis lupus*) and spotted hyena (*Crocuta crocuta*) weighing 20–60 kg can reduce a 70+ kg carcass to scraps of skin and bone extremely rapidly, certainly in under an hour [61,62]. If similar-sized carnivorous theropods had the same ecological impact in the Late Cretaceous, this would have severely reduced the availability of the most common carcass size available to larger carnivorous species. Although a fully grown *T. rex*, being such a large carnivore, may have dominated carcasses once it had arrived [63,64], there would have been a limited number of carcasses persisting in the environment to support such a giant obligate scavenger. Even without the impact of small theropods, predicted travel speeds and detection ranges indicate that carcasses would have rotted by the time an adult *T. rex* was typically able to find them. Furthermore, whereas our analysis has only considered potential dinosaur competitors, *T. rex* may also have competed with other species such as giant azhdarchid pterosaurs for access to carcasses [12,65], suggesting that Late Cretaceous North America would have been even less likely to support a giant obligate terrestrial scavenger than we have predicted. Decomposers would also have played their part in further reducing the amount of meat available to vertebrate scavengers [7]. In addition, our analyses made the conservative assumption that theropod speed increases with size. Recent studies on theropod locomotion, however, suggest that smaller species may in fact have been faster and more mobile [66,67]. This is consistent with patterns of home range size in modern carnivores, where the largest home ranges are found in the intermediate body size range [68]. If movement rates of smaller species were in fact higher than we have assumed, the difference in search rates would be even more extreme.

The question of whether dinosaurs were endothermic or ectothermic remains one of the great unresolved controversies of palaeontology, and has important implications for reconstructing aspects of dinosaurian trophic ecology such as metabolic requirements and predator : prey ratios [69–71]. Our comparison of the search rates of theropod dinosaurs is sensitive to changes in relative abundance, speed of movement and detection distances. While these variables would be affected by metabolic rate, our main conclusions regarding competition between theropods would not be affected by their thermoregulatory status if we assume that all theropods in the *T. rex* community had similar metabolic rates, a plausible assumption following several lines of evidence (e.g. phylogeny, shared possession of feathers or protofeathers, bipedal stance [27,70]). If theropods were ectotherms, we would expect *T. rex* to be able to tolerate longer periods of fasting, but we might also expect it to move more slowly and have a lower search rate [17]. We might expect ectothermic theropods to obtain higher densities for a given density of prey/carcasses, but this would also lead to higher levels of competition for each carcass. These tradeoffs therefore suggest that our conclusions about the trophic status of *T. rex* should hold true whether it was endothermic or ectothermic.

In a wider ecological context, it should not be surprising that a fully grown *T. rex* cannot be realistically interpreted as an obligate scavenger. Large-bodied theropods (e.g. neoceratosaurs, carnosaurs, megalosaurs, tyrannosauroids) are characteristic members of all well-studied diverse Jurassic and Cretaceous dinosaur assemblages, and almost all of these species other than *T. rex* have been interpreted uncontroversially as active terrestrial predators [24]. The only large-bodied theropods that probably had a markedly different nutritional ecology are the fish-eating spinosaurs, which unlike *T. rex* have been reconstructed on the basis of several independent lines of evidence (functional morphology, isotope signatures and stomach contents [72–74]). Several Jurassic and Cretaceous predator guilds (e.g. Morrison Formation, Bahariya Formation) contained multiple lineages of extremely large sympatric predatory theropods [24,75], for which direct competition for prey must have been mitigated by morphologically or ecologically mediated niche partitioning [56]. In contrast, tyrannosaurids are the only large-bodied theropods in Late Cretaceous North America, with *T. rex* as the only giant carnivore (see [34] for tyrannosaurid body masses and growth rates). Since the *T. rex* community also contained a wide range of large-bodied potential prey species, it can be considered extremely unlikely on comparative ecological grounds that this system for some reason lacked a similar theropod ‘super-predator’.

Many extant mammalian carnivores feed in groups, especially in open and highly competitive ecosystems, and recent work on Pleistocene carnivores suggests that sociality may make species more effective at defending scavenged carcasses from competitors [64,76]. If intra-guild competition was similarly high in Late Cretaceous terrestrial ecosystems, we might also expect selection for social grouping behaviour in carnivorous theropods. Interestingly, although reconstructing social behaviour for extinct species in the fossil record is an extremely difficult process, dromaeosaurs have often been interpreted as social carnivores (‘pack hunters’) on the basis of *Deinonychus*–*Tenontosaurus* fossil assemblages [77] (although see [78]), and there is also some taphonomic evidence to suggest gregarious behaviour in the small tyrannosaurid *Albertosaurus* [79].

What can our analysis tell us about other aspects of the predatory behaviour of *T. rex* and other extremely large theropods? Clearly, *T. rex* would have been able to dominate almost all other terrestrial predators at any time or any place. However, given its large mass and the fact that it would have been vastly outnumbered by smaller theropods, it was probably a poor competitor in indirect scramble competition. We have evaluated the potential impact of intra- and interspecific competition between sympatric theropods on scavenging opportunities for *T. rex*, but similar arguments could also be applied to its ability to find hatchling or juvenile dinosaurs or other sources of food if these had an unpredictable and ephemeral distribution, even though these are sometimes interpreted as possible specialist tyrannosaurid prey items [80]. Given its physical dominance, being one of the largest known theropods [36], *T. rex* would have been better able to compete for more predictable food resources, and as a large prey specialist it would have had a unique position in its guild at being able to effectively hunt herbivorous dinosaurs that were far larger than would be available to other theropod species. Indeed, although pack hunting may have allowed dromaeosaurs to target larger prey species, taphonomic evidence suggests that small Cretaceous deinonychosaurian theropods probably hunted relatively small-bodied herbivorous dinosaurs such as basal neoceratopsians or hypsilophodontids, or dinosaur hatchlings [31,57,77], rather than the larger hadrosaurs, neoceratopsians or titanosaurs. As an active large prey specialist, feeding on herbivores of similar or greater mass, *T. rex* would have had feeding habits consistent with prey size selection patterns found in extant mammalian carnivores [81,82]. We propose that this is the most likely feeding strategy for *T. rex*. Future research into the energetic and behavioural constraints on this extreme giant predator might also help to better understand the evolutionary constraints on carnivory.
